# Development of Nanocomposite Coatings

**DOI:** 10.3390/nano12244377

**Published:** 2022-12-08

**Authors:** Zulfiqar A. Khan, Mian H. Nazir, Adil Saeed

**Affiliations:** 1 NanoCorr, Energy & Modelling (NCEM) Research Group, Department of Design & Engineering, Bournemouth University, Talbot Campus, Poole BH12 5BB, UK; 2 Faculty of Computing Engineering and Sciences, University of South Wales, Treforest Campus, Pontypridd CF37 1BF, UK

This Special Issue in *Nanomaterials*, “Development of Nanocomposite Coatings”, was set up with the aim to provide authors with an opportunity to showcase their latest developments in this field. We therefore welcomed research articles and reviews papers for possible publication, by invitation only, on nanocomposite coatings. Nanocomposite coatings have wide-ranging applications, especially in the context of cavitation, including corrosion, tribology, machine elements, components, complex interacting systems, and fluid flow. We have witnessed an increased need for applications addressing key global and industrial challenges, including those relating to the energy efficiency, reliability, sustainability, and durability of systems and machines. These components and systems are often deployed in harsh operating environments and conditions, for example, very high or subzero temperatures, extreme pressures, very high loading requirements, corrosive environments, and starved lubrication. To solve these issues, novel and innovative approaches are needed. These solutions include optimisation of surfaces and interfaces through surface modifications and coatings. The development and application of nanocoatings and nanocomposite coatings is a relatively new field, and studies in this area are underway.

Design and engineering processes and techniques focused on the development of nanocomposite coatings have provided significant opportunities to address the above-mentioned key issues. In turn, researchers around the globe have been actively engaged in studying several types of nanocomposites with the aim of developing durable and energy-efficient coatings. This offers several academic and industrial benefits.

Experimental results and numerical models of alumina, silicon carbide, zirconia, and graphene nanocomposite coatings have been previously presented and published. These coatings have been experimentally investigated by employing a micro-friction reciprocating bench-top test machine. Tests and load configurations with three types of experimental media have been conducted as: uncontaminated oil, contaminated oil with 5% wt. sea water, and contaminated sea salt with 5% wt. and 10% wt. water [1,2,3,4].

The cathodic blistering model Khan-Nazir I was developed and reported in [5] and is provided below in Equation (1).

(1) $$\frac{6\left(1-v_{c}^{2}\right)}{E_{c}h^{3}}\left[M_{c}^{2}+{\left(0.2\left(1+v_{c}\right)\sqrt{\frac{1}{0.2\left(1+v_{c}\right)+0.2\left(1-v_{c}^{2}\right)}\left(\frac{p}{p_{cr}}-1\right)}\right)}^{2}\right]$$

where $v_{c}$ = Poisson’s ratio of coating; E_c_ = elastic modulus of coating; h = thin film, coating thickness; M_c_ = blistering-induced film–substrate system bending moment; *p* = blister pressure; $p_{cr}$ = critical pressure [2].

The coating failure model Khan-Nazir II has been extended to nanocomposite coating failure subject to wear–corrosion as a mechano-wear model [6], as shown in Equation (2).

(2) $$\overset{↔}{J}=\frac{F\rho }{KA_{s}\;t}\left(V-\left(K_{v}\sum W\right)-S\right)$$

where $\overset{↔}{J}$ = corrosion current density; *F* = Faraday’s constant; *ρ* = coating density; *K* = specimen material’s electrochemical equivalent; *A_s_* = area of the specimen; *t* = time; V = wear–corrosion volume loss; $K_{v}$ = wear rate; W = Archard factor density; *S* = synergistic factor [7]. Lubrication modelling with wear–corrosion and mechano-wear equations were added to investigate the influences of the intrinsic microstructural properties of nanocomposite coatings, for instance, the porosity and surface stresses, on the coefficient of friction (CoF) [1].

(3) $$µ=\left(u\eta_{0}\pi {\left(4PR/\pi L\left(\left(1-v_{1}^{2}\right)/E_{1}+\left(1-v_{2}^{2}\right)/E_{2}\right)\right)}^{2}\right)/\left(Ph_{oil\infty }\right)$$

where the coefficient of friction is denoted by µ, while the normal force is represented by *P*, and the radius of the steel ball is taken as *R*; *L* is the axial length of the ball–coating contact; $v_{1}$ and $E_{1}$ are the Poison ratio and Young’s moduli of the coating; $v_{2}$ and $E_{2}$ are the Poison ratio and Young’s moduli of substrate, respectively; $\eta_{0}$ is the viscosity of the bulk lubricant; $h_{oil\infty }$ is the remaining thickness of the oil in the vicinity of the asperity contact.

Special attention is paid in this issue to experimental methods for analysing the corrosion degradation of nanocomposite coatings. For example, two major types of coating deteriorations are (i) cathodic delamination and (ii) cathodic blistering, including wear–corrosion dilapidation, which are commonly seen in coatings subject to wear and corrosive environments. These failure mechanisms initiate and propagate from pre-existing defects which exist in coatings; this in turn facilitates corrosive species diffusion from an external electrolyte [8]. When cathodic delamination is considered, the parting of the film is mainly driven by the cathodic reaction in a localised corrosion cell [7].

Important improvements suggested in ref. [6] can be incorporated into the existing modelling techniques to improve the failure predictions of nanocomposite coatings, for example: micro-crack effects [9,10,11], multi-layer film effects [12], potential distribution within delaminating region [13], and the nature of film–substrate bonding [14].

Micro- and nanosensor development for motioning the degradation rate of nanocomposite coatings has always been an important research area. Some vital research in sensing technology is mentioned herein [15].

A recent study synthesised magnetic chitosan nanocomposites by employing an established ultrafast US irradiation strategy, which presented good crystallinity, a high Ms range, and high surface area [16]. Likewise, Chitosan-TiO_2_ coatings, which have various concentrations of TiO_2_NPs, were produced, and their thermal, antimicrobial, and physicochemical characteristics were methodically categorised [17]. In another study, fabrication of nanostructured Co-Pb composite anodes was achieved by employing ARB. The results of this investigation demonstrate that the ARB process enhances the mechanical attributes of Co-Pb anodes [18]. One novel surface treatment process used to develop nanocoating is plasma electrolytic oxidation (PEO). The PEO process causes transformations of microstructures within coatings; a reduction in residual stresses within coatings takes place, enhancing coating homogeneity [19].

A Nb alloying layer over a TC4 substrate was developed via various HCPEB irradiation pulses, which led to improved surface performance [20]. Likewise, another study explored WN/MeN (Me = Cr, Zr, Mo, Nb) nanocomposite multilayered coatings deposited by CA-PVD via analysing the microstructures, wear behaviour, friction performance, and mechanical properties of WN-based multilayers [21]. Another research work demonstrates a one-pot, hydrothermal, template-free method for the successful synthesis of α-Fe_2_O_3_ (hematite) and Fe_3_O_4_ (magnetite) [22].

Recent developments in nanocomposite coatings, real-time monitoring, failure prediction modelling, and future systems have been reported in this Special Issue. We hope that researchers, scientists, academics, and industry professionals will find this Special Issue inspiring for the further enhancement of design and development of nanocomposite coatings. We look forward to receiving further contributions on the impactful and fascinating theme herein to this Special Issue.
